# An Enhanced User Authentication and Key Agreement Scheme for Wireless Sensor Networks Tailored for IoT

**DOI:** 10.3390/s22228793

**Published:** 2022-11-14

**Authors:** Pooja Tyagi, Saru Kumari, Bander A. Alzahrani, Anshay Gupta, Ming-Hour Yang

**Affiliations:** 1 Department of Mathematics, Chaudhary Charan Singh University, Meerut 250004, India; 2 Faculty of Computing and Information Technology, King Abdulaziz University, Jeddah 21589, Saudi Arabia; 3 Department of Computer Science and Engineering, HMR Institute of Technology and Management, New Delhi 110036, India; 4 Department of Information and Computer Engineering, Chung Yuan Christian University, Taoyuan 320314, Taiwan

**Keywords:** key agreement, smart card, user authentication, wireless sensor networks

## Abstract

A security protocol for wireless transmission is essential to defend sensitive information from malicious enemies by providing a variety of facilities such as privacy of the user’s information, secure session key, associated authentication, and user-repeal facility when a person’s authorizations are suddenly disclosed. Singh et al. proposed an improved user authentication and key agreement system for wireless sensor networks (WSNs). Authors are sure that their protocol is secure from various attacks. Here, we find several security pitfalls in their scheme, such as an offline password-guessing attack, failure to protect the session key, and a man-in-the-middle attack. To remove the identified pitfalls found in Singh et al.’s scheme, we design an enhanced authentication scheme for WSNs tailored for IoT. We prove the reliability of our proposed protocol using the real or random (RoR) model. We also evaluate the proposed scheme with the associated schemes and show its superior efficacy as compared to its counterparts.

## 1. Introduction

A wireless Sensor Network (WSN) consists of sensors or sensor nodes and plays a vital role in the Internet of Things (IoT) applications. The sensor nodes can be used at sensitive places in an unplanned or planned way. These kinds of nodes have the capacity to collect data from their neighboring fields, after which they send the data to nearby base stations (BSs), which process the received data for decision-making. Sensor nodes can communicate with each other via wireless radio communications. In WSNs, the BS (referred to as the gateway node (or GW-node)) is the most effective node, whereas sensors are the least effective nodes in regard to battery power, memory space, and computational ability.

WSNs can be utilized in different unattended fields, such as the army, climatic, medical, and agriculture, for goal monitoring, battleground vigilance, and invader identification. WSNs can also be deployed in various IoT applications such as smart homes, smart supply-chain management, smart cities, smart grids, smart traffic management, and industrial Internet. Due to the unattended surroundings of sensor nodes, an adversary has the ability to immediately capture a sensor node from the goal-tracking area. For this reason, an adversary has a possibility to at once seize a sensor node from the goal field and extract all of the data from its reminiscence, as nodes are not usually tamper-resistant because of their low cost.

The requirement to protect the data stored in WSNs is a crucial issue. Here we discuss some scenarios which necessitate a user verification protocol in WSNs. In WSNs, an intruder can create a bug in the network and can disturb or discontinue the commuted texts. Numerous crucial operations in WSNs, along with the utilization of the battleground surveillance and e-health services, rely on actual data to obtain which the users establish a direct connection with the sensor nodes with the help of the base station. Thus, in applications requiring actual data, the user’s authenticity is verified by the sensor nodes and the BS, which requires setting up a secret session key among the users and the accessed nodes so that no intruder can obtain the crucial data from the sensor nodes. Due to this motive, user authentication and key agreement protocols in WSNs are important research fields.

### Motivation and Contribution

WSNs’ environment is challenging due to their resource-constrained nature and security requirements. The same is true for any IoT application due to its dependency on WSNs. Authentication and key agreement schemes are utilized to handle the application layer in WSNs/IoT. In these schemes, balancing efficiency and security is a big challenge. In this regard, research on establishing authentication and key agreement is in the developing stage. While reviewing some literature on authentication and key agreement scheme for WSN, we came across an article in which we felt scope for improvement. The contributions of this article are as follows:

- We analyze an authentication and key agreement scheme for WSNs and point out its flaws.
- As an enhancement of the analyzed scheme, we propose an authentication and key agreement scheme for WSNs tailored for the IoT.
- We have tried to achieve the maximum possible security features while keeping the minimum possible computational load.

## 2. Related Work

In 2009, Das [1] proposed two-factor user authentications in WSNs. The author claims that the proposed scheme resists many attacks in WSNs, but it suffers from denial-of-service and node compromise attacks. In 2011, Yeh et al. [2] worked on Das’s scheme and proposed an authentication protocol for WSNs using elliptic curve cryptography. Mutual authentication is important to prove the legitimacy of each party, and Yeh et al. [2] found that the scheme in [1] does not provide mutual authentication; they also found that the protocol in [1] suffers from insider attacks, user impersonation attacks, and no provision for changing updating passwords.

In 2013, Xue et al. [3] proposed a temporal credential-based mutual authentication scheme for WSNs. In 2014, Turkanovic et al. [4] suggested a user-mutual authentication key agreement protocol for heterogeneous ad hoc WSNs. This scheme concentrates on the Internet of things (IoT) notion. In 2015, Jiang et al. [5] found some security flaws in the protocol [3] and proposed an improvement of [3]. Jiang et al. found that the scheme in [3] suffers from insider attacks, weak stolen smart card attacks, identity guessing attacks, and tracking attacks. In 2015, He et al. [6] proposed mutual authentication and key agreement protocol for WSNs. He et al. [6] found that the scheme in [3] cannot withstand the security parameters, and protocol [3] suffers from offline password guessing attacks, user impersonation attacks, and sensor node impersonation attacks. He et al. [6] found that the scheme in [3] cannot provide legitimacy of the user.

In 2016, Kumari et al. [7] pointed out that the scheme in [6] has many disadvantages. Kumari et al. [7] showed that protocol [6] suffers from offline password-guessing attacks, session-specific temporary information attacks, the absence of password-changing facilities, and the absence of unauthorized login detection, and it does not provide legitimacy to the user. Kumari et al. [7] proposed a mutual authentication and key agreement scheme for WSNs using chaotic maps. In 2016, Jiang et al. [8] worked on the scheme in [6], and after analysis, they showed that the scheme in [6] fails to provide anonymity for the user. Jiang et al. [8] found that in the protocol [6], an adversary can easily track the user, and also, this scheme cannot stand with stolen smart card attacks. Jiang et al. [8] proposed an untraceable temporal-credential-based authentication scheme for WSNs.

In 2016, Farash et al. [9] noticed that the protocol in [4] does not resist man-in-the-middle attacks, the disclosure of the session key, sensor node impersonation attacks, and the disclosure of secret parameters. Farash et al. [9] also showed that the scheme in [4] does not provide sensor node anonymity, and any adversary who wants to track the user can easily do so. To remove the weakness, Farash et al. [9] proposed an enhanced scheme. In 2016, Amin and Biswas [10] found that the protocol in [4] undergoes offline password-guessing attacks, offline identity-guessing attacks, smart card theft attacks, user impersonation attacks, sensor-node impersonation attacks, and inefficient authentication phase. Amin and Biswas [10] also gave an authentication method after removing the weakness of the scheme in [4], and they claimed the enhanced security of their scheme over protocol in [4]. Amin and Biswas used BAN logic for formal security analysis of the proposed protocol.

In 2016, Amin et al. [11] showed that the scheme in [9] is vulnerable to smart card stolen attacks, offline password-guessing attacks, new smart issue attacks, user impersonation attacks, and known session-specific temporary information attacks. Amin et al. [11] found that the protocol in [9] does not provide user anonymity, and also the secret key of the gateway node is not safe in this protocol. To overcome the disadvantages of the scheme in [9], Amin et al. [11] put forward an improved version of WSNs. In 2016, Chang and Le [12] showed that protocol in [4] is vulnerable to impersonation attacks with node capture, stolen smart card attacks, sensor node spoofing attacks, and stolen verifier attacks, and it fails to ensure backward secrecy. To remove these security issues, Chang and Le [12] proposed an advanced scheme.

In 2017, Wu et al. [13] revealed that the scheme in [10] suffers from sensor capture attacks, session key leakage attacks, user forgery attacks, gateway forgery attacks, and sensor forgery attacks. Wu et al. [13] showed that in the scheme [10], the adversary could track the user easily, and this does not provide mutual authentication between parties. To remove the disadvantages in [10], Wu et al. [13] proposed an authentication scheme for multi-gateway-based WSNs. In 2017, Wu et al. [14] found that the scheme in [5] has many security pitfalls, such as offline password-guessing attacks, user forgery attacks, de-synchronization attacks, and a lack of strong forward security. Wu et al. [14] recommended a stepped-forward version of the protocol in [5]. They claimed their protocol to be secure. In 2017, Dhillon and Kalra [15] proposed multi-factor remote user authentication and key agreement scheme for IoT environments. They said that their proposal is to be defendable against all prospected threats.

In 2018, Amin et al. [16] designed a robust patient monitoring system using wireless medical sensor networks. They are sure that their protocol is secured against obvious violations. They used BAN logic to confirm the mutual authentication feature for their suggested protocol. In 2018, Jangirala et al. [17] designed an authentication and key agreement protocol for the industrial Internet of things. In the scheme of [17], they used the fuzzy extractor method for biometric authentication by the user’s smart card. In 2018, Li et al. [18] pointed out that the protocol in [8] cannot resist known session-specific temporary information attacks and clock synchronization, and this scheme is not applicable to IoT environments. To improve the scheme in [8], Li et al. [18] suggested a three-factor anonymous authentication scheme for WSNs. In 2018, He et al. [19] found that the scheme in [12] suffers from sensor capture attacks. They gave an improved version of [12].

In 2019, Gupta et al. [20] designed an anonymous user authentication and key-establishment scheme for wearable devices. They used BAN logic to justify mutual verification among the gateway/cellular terminal and the wearable gadget of the sensor. In 2019, Ghani et al. [21] proposed an IoT-based scheme for WSNs using a symmetric key. In 2020, Lee et al. [22] discovered that the protocol in [15] is vulnerable to a stolen mobile device attack and a user impersonation attack, and it lacks a provision for the agreement of the session key. In 2021, Mall et al. [23] proposed a physically unclonable function (PUF) based authentication protocol for drone-enabled WSNs. This protocol conducts communication among devices and the cloud by the relocatable drone.

In 2021, Chen et al. [24] suggested a group key agreement protocol for IoT. In this scheme, they introduce an entity known as the device manager. Device managers connect IoT devices with blockchain networks. In 2021, Chen and Liu [25] suggested a three-factor scheme for the IoT that used biological information. They proved their protocol in both a formal and informal manner. In 2021, Ali et al. [26] designed an ECC-based protocol for vehicle-to-vehicle communication in VANETs. In 2021, Sadri and Asaar [27] showed that the scheme in [21] has many security pitfalls, such as user impersonation attacks, malicious gateway attacks, and traceability attacks. To remove weaknesses found in [21], Sadri and Asaar [27] suggested a hash-based scheme for WSNs in IoT with forward secrecy. They analyzed their protocol with both formal and informal methods. In 2021, Rangwani et al. [28] proposed a three-factor scheme for the Industrial Internet of Things (IIoT). They also verified their scheme in both formal and informal ways. In 2021, Nashwan [29] designed a scheme for healthcare IoT. In this scheme, mutual authentication between all nodes is verified using BAN logic. 

In 2022, Tanveer et al. [30] suggested a resource-capable scheme for the Industrial Internet of Things (IIoT). The authors claimed that their scheme is suitable for resource-constrained smart devices. In 2022, Kumar et al. [31] designed an RFID-based scheme using PUF for vehicular cloud computing. In 2022, Wu et al. [32] suggested a scheme that depends on a symmetric encryption algorithm and fog computing in the Internet of vehicles. In 2022, Li et al. [33] proposed a protocol for fog-enabled social internet of vehicles.

In 2016, Singh et al. [34] proposed a scheme to resolve the weaknesses of the protocol in [4]. Singh et al. claimed their scheme to be more secure and efficient for a real application environment. However, we show that the protocol in [34] has many security issues. The scheme in [34] suffers from many attacks like offline password-guessing attacks, man-in-the-middle attacks, and attacks on the session key.

### Organization

In Section 3, we review Singh et al.’s scheme. The cryptanalysis of Singh et al.’s scheme is shown in Section 4. Section 5 describes our enhanced scheme. Section 6 explains the security analysis of the proposed scheme in both a formal and informal manner. Section 7 contains a comparison of the proposed scheme with some related schemes. The conclusion is in Section 8.

## 3. Review of Singh et al.’s Scheme

**Figure 1:**
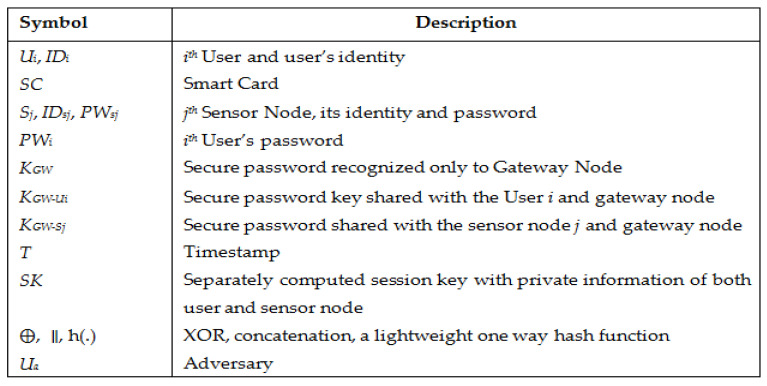
Notations and description.

Firstly, we write the notations and their explanations used in this paper in Figure 1.

### 3.1. Registration Phase

**Figure 2:**
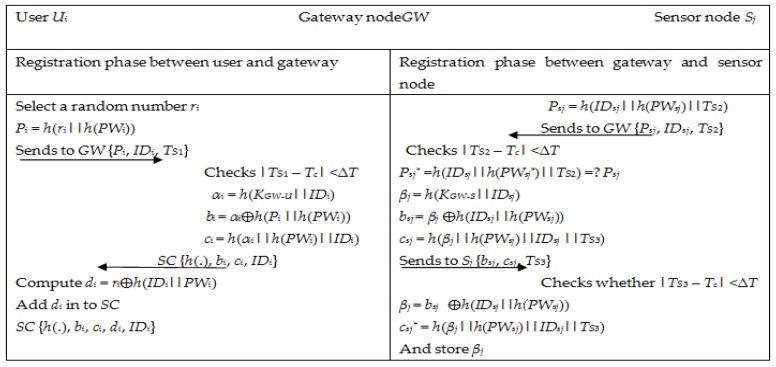
Registration phase of Singh et al.’s scheme [34].

The system of registration begins after the placement of sensor nodes in the application space. The registration phase is split into two sub-phases. Phase one is between a user and the gateway, and phase two is between the sensor node and the gateway. Figure 2 illustrates all two stages.

#### 3.1.1. Registration Between User and Gateway

Identity (*ID_i_*) and secure password (*PW_i_*) are provided to every user. User identity and password hash value are saved in the gateway node. At first, the gateway selects a random key *K_GW-__U_*. With this key, *GW* can communicate with the user. The gateway further selects a different key *K_GW-__S_*. With this particular key, *GW* can communicate with sensor nodes. The procedure for this phase is as follows:

Step-1: User *U_i_* selects a random number *r_i_* and computes *P_i_* = *h*(*r_i_*||*h*(*PW_i_*)).

Step-2: User generates time stamp *T*_s1_ and sends {*P_i_*, *ID_i_*, *T*_s1_} to *GW* through a protected channel.

Step-3: Following the received message, the gateway verifies the legitimacy of a time stamp. 

If |*T_S_*_1_ − *T_c_*| < ∆*T* exists, then gateway calculates.

- *α_i_* = *h*(*K_GW-U_* ||*ID_i_*)*;*
- *b_i_* = *α_i_* ⨁ *h*(*P_i_* ||*h*(*PW_i_*));
- *c_i_* = *h*(*α_i_*||*h(PW_i_*)||*ID_i_*);

Step-4: Gateway customizes *SC* with {*h*(.), *b_i_*, *c_i_*, *ID_i_*} and conveys to the user through a protected channel.

Step-5: User adds *d_i_* = *r_i_* ⨁ *h*(*ID_i_*||*PW_i_*) into *SC*. Now *SC* contains {*h*(.), *b_i_*, *c_i_*, *d_i_*, *ID_i_*}.

#### 3.1.2. Registration Between Sensor node and gateway

Every sensor node has an identity (*ID_sj_*) and a protected password (*PW_sj_*). The identity and the hash value of the password for sensor node *S_j_* are also saved in the gateway. The phase consists of the following steps:

Step-1: Sensor node *S_j_* computes *P_sj_* = *h*(*ID_sj_*||*h*(*PW_sj_*)||*Ts*_2_) with its *ID_sj_* and *PW_sj_*.

Step-2: Sensor node dispatch message {*P_sj_*, *ID_sj_*, *Ts*_2_} to the gateway.

Step-3: When information is received, the gateway confirms the validation of a time stamp. If |*Ts*_2_ − *T_c_*| < ∆*T,* then it moves ahead or else sends non-acceptance text to the sensor node.

Step-4: With secret key *K_GW-S_*, *GW* calculates the following values:

- *β_j_* = *h*(*K_GW-S_*||*ID_sj_*);
- *b_sj_* = *β_j_* ⨁ *h*(*ID_sj_*||*h*(*PW_sj_*));
- *c_sj_* = *h*(*β_j_*||*h*(*PW_sj_*)||*ID_sj_*||*Ts*_3_);

Step-5: *GW* sends {*b_sj_*, *c_sj_*, *Ts*_3_} to the sensor node through a non-private channel.

Step-6: After confirmation of obtaining the data, the sensor node verifies the legitimacy of a time stamp. If |*Ts*_3_ − *T_c_*| < ∆*T*, then move ahead to the succeeding step or else deliver a non-acceptance message to *GW*. 

Step-7: Sensor node calculates *β_j_* = *b_sj_* ⨁ *h*(*ID_sj_*||*h*(*PW_sj_*)) and checks c_sj_^*^ = *h*(*β_j_*||*h*(*PW_sj_*)||*ID_sj_*||*Ts*_3_) is equal to *c_sj_*; after that, saves *β_j_* into its memory or else sends a failure message to *GW*.

### 3.2. Login Phase

**Figure 3:**
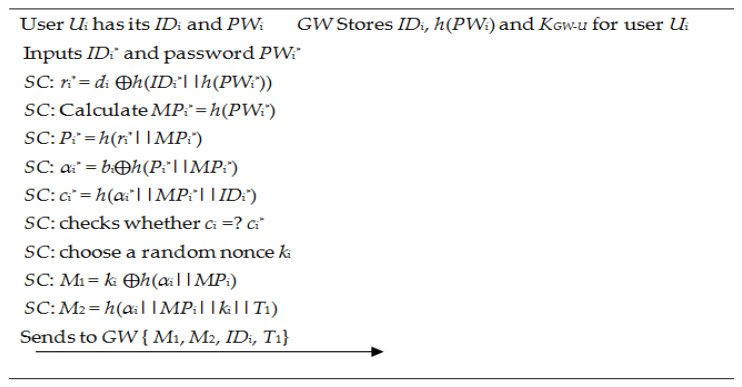
Login phase of Singh et al.’s scheme [34].

After the registration phase, the connection is established between the user and *S_j_* via the *GW* node. Figure 3 describes the work-flow of the login phase. The steps are as follows:

Step-1: User *U_i_* inserts his/her card into the insertion area and enters his/her ID_i_^*^ and password PW_i_^*^.

Step-2: *SC* calculates r_i_^*^ = *d_i_* ⨁ *h*(ID_i_^*^||PW_i_^*^) with the saved value of *d_i_*. Then it calculates MP_i_^*^ = *h*(PW_i_^*^) and *P_i_* = *h*(r_i_^*^||MP_i_^*^).

Step-3: Then, smartcard calculates α_i_^*^ = *b_i_* ⨁ *h*(*P_i_*||MP_i_^*^).

Step-4: *SC* calculates one more time c_i_^*^ = *h*(α_i_^*^||MP_i_^*^||ID_i_^*^) and verifies whether the original *c_i_* or computed c_i_^*^ are the same. If it is not equal, then the login progress will be terminated.

Step-5: If the entered password is exactly the same, the user selects an arbitrary number *k_i_* and calculates *M*_1_ = *k_i_* ⨁ *h*(*α_i_*||*MP_i_* ) and *M*_2_ = *h*(*α_i_*||*MP_i_*||*k_i_*||*T*_1_).

Step-6: User sends {*M*_1_, *M*_2_, *ID_i_*, *T*_1_} to *GW* through an open channel.

### 3.3. Authentication and Key Agreement Phase

**Figure 4:**
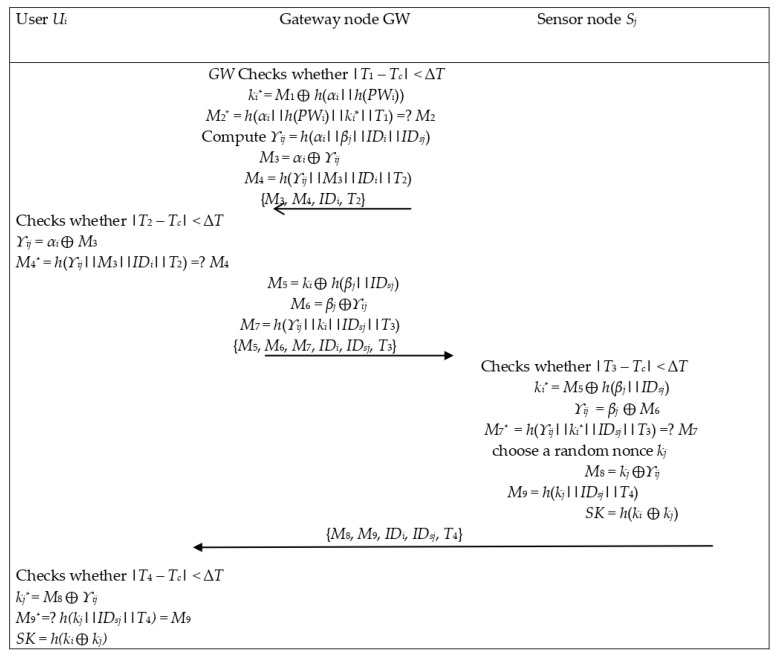
Authentication phase of Singh et al.’s scheme [34].

Mutual confirmation among all groups is made after the success of the login phase. This procedure is performed in the authentication and key agreement phase. It takes three steps. The first one is for the user’s authority confirmation through *GW*. The second one represents the *GW’s* lawfulness confirmation by the user and the sensor node. Moreover, the third one is for the user to verify the authentication of the sensor node. The focus of this phase is providing a session key between the user and the sensor node. This phase is illustrated in Figure 4. The whole authentication and key agreement phase is discussed in the following steps.

Step-1: As the gateway obtains a message {*M*_1_, *M*_2_, *ID_i_*, *T*_1_} from the user *U_i_*, the gateway verifies the time stamp’s validity by calculating |*T*_1_ − *T_c_*| < ∆*T.* If it is found valid, *GW* again calculates the upcoming step or else sends a failure message to *U_i_.*

Step-2: With the help of *h*(*PW_i_*), as per the accepted *ID_i_*, the gateway calculates k_i_^*^ = *M*_1_ ⨁ *h*(*α_i_*||*h*(*PW_i_*)) and after that calculates its own version of *M*_2_^*^ = *h*(*α_i_*||*h*(*PW_i_*)||k_i_^*^||*T*_1_) and compares it with the received *M*_2_. In case these are the same, then *GW* validates the user *U_i_* or else sends a failure text to the user.

Step-3: Once the validation of the user is completed, *GW* calculates *ϒ_ij_* = *h*(*α_i_*||*β_j_*||*ID_i_*||*ID_sj_*), *M*_3_ = *α_i_* ⨁ *ϒ_ij_*, and *M*_4_ = *h*(*ϒ_ij_*||*M*_3_||*ID_i_*||*T*_2_) and sends {*M*_3_, *M*_4_, *ID_i_*, *T*_2_} to the user; here, *T*_2_ represents *GW’s* time stamp.

Step-4: After obtaining {*M*_3_, *M*_4_, *ID_i_*, *T*_2_}, the user verifies whether |*T*_2_ *− T_c_*| < ∆*T* and after that calculates its own version of *ϒ_ij_* = *α_i_* ⨁ *M*_3_ and *M*_4_^*^ = *h*(*ϒ_ij_*||*M*_3_||*ID_i_*||*T*_2_). The user checks whether *M*_4_ =? *M*_4_^*^. If both are the same, then gateway authorization by the user *U_i_* holds. If not, the user discontinues the procedure by sending a failure message to *GW*.

Step-5: At time *T*_2_ when message is sent to user *U_i_*, *GW* calculates *M*_5_ = *k_i_* ⨁ *h*(*β_j_*||*ID_sj_*), *M*_6_ = *β_j_* ⨁ *ϒ_ij_*, and *M*_7_ = *h*(*ϒ_ij_*||*k_i_*||*ID_sj_*||*T*_3_) after that forwards {*M*_5_, *M*_6_, *M*_7_, *ID_i_*, *ID_sj_*, *T*_3_} to sensor node *S_j_*.

Step-6: When a message is received from *GW*, now *S_j_* verifies if |*T*_3_ *− T_c_*| < ∆*T* then further calculates owned version of k_i_^*^ = *M*_5_ ⨁ *h*(*β_j_*||*ID_sj_*) by using saved *β_j_* and after this calculates its own version of *ϒ_ij_* = *β_j_* ⨁ *M*_6_ and *M*_7_^*^ = *h*(*ϒ_ij_*||k_i_^*^||*ID_sj_*||*T*_3_) and compares *M*_7_^*^ with *M*_7_. It checks the values; if both are equal, then *GW* is verified by *S_j_*, or else *S_j_* transmits a failure text to *GW*.

Step-7: When authentication of *GW* is complete, *S_j_* chooses a random number *k_j_* and calculates the session key, which is *SK* = *h*(*k_i_* ⨁ *k_j_*).

Step-8: In the end, thesensor node *S_j_* calculates *M*_8_ = *k_j_* ⨁*ϒ_ij_* and *M*_9_ = *h*(*k_j_*||*ID_sj_*||*T_4_*) and transmits {*M*_8_, *M*_9_, *ID_i_*, *ID_sj_*, *T*_4_} to user *U_i_*.

Step-9: When the text is received from sensor node *S_j_*, the user verifies the legality of the time stamp |*T*_4_ *− T_c_*| < ∆*T*a and verifies the validity of *S_j_* by calculating its own version of *k_j_* = *M*_8_ ⨁*ϒ_ij_* and *M*_9_^*^ = *h*(*k_j_*||*ID_sj_*||*T*_4_) and after that analyzes *M*_9_^*^ with the accepted *M*_9_. It checks if both are the same and furthermore calculates the session key as *SK* = *h*(*k_i_* ⨁ *k_j_*), then, as a result, efficiently ends the authentication phase.

## 4. Cryptanalysis of Singh et al.’s Scheme 

### 4.1. Insider Attack

Suppose an insider at *GW* can obtain a user smart card and access the information saved in *SC* {*h*(.), *b_i_*, *c_i_*, *d_i_*, *ID_i_*}. In the registration phase, when *U_i_* submits {*P_i_*, *ID_i_*}, the insider guesses the password *PW_i_* and finds *r_i_* in the following way:

*r_i_ = d_i_* ⨁ *h*(*ID_i_||h(PW_i_*))

after that calculates *P_i_^#^* = *h*(*r_i_*||*h(PW_i_)*) and checks whether *P_i_* = ? *P_i_^#^*

The insider guesses the password till he/she achieves the correct password.

### 4.2. Offline Password Guessing Attack

Secret parameters saved into smart card are {*h*(.), *b_i_*, *c_i_*, *d_i_*, *ID_i_*}

An adversary *U_a_* can do guesswork PW_i_^*^ for the password, and now computes *r_i_^#^* = *d_i_* ⨁*h*(*ID_i_*||*PW_i_^#^*)

Then, the adversary finds the value of *P_i_^#^* from *P_i_^#^* = *h*(*r_i_^#^*||*h*(*PW_i_^#^*)). The adversary computes the value 

*α_i_^#^* = *b_i_* ⨁ *h*(*P_i_^#^*||*h(PW_i_^#^*)). Then, the adversary computes *c_i_^#^* = *h*(*α_i_^#^*||*h(PW_i_^#^*)||*ID_i_*) and checks whether *c_i_*^#^ =? *c_i_*. If it holds, the adversary obtains an exact password *PW_i_*. In any other case, the adversary repeats the process.

### 4.3. Lack of User Anonymity

In Singh et al.’s scheme, messages {*M*_1_, *M*_2_, *ID_i_*, *T*_1_**},** {*M*_3_, *M*_4_, *ID_i_***,** *T*_2_**},** and {*M*_8_, *M*_9_, *ID_i_***,** *ID_sj_***,***T*_4_**}** directly involve the identity *ID_i_* of a valid user *U_i_* in plain text. By spy monitoring the messages, an adversary recognizes *ID_i_*. Subsequently, Singh et al.’s scheme does not hold the user anonymity property.

### 4.4. Man-In-The-Middle Attack

During the attack, an adversary *U_a_* tries to know the actual session key.

1. When the user *U_i_* transmits the login message {*M*_1_, *M*_2_, *ID_i_*, *T*_1_} to *GW* via a pubic channel, the adversary *U_a_* intercepts the message and plunders the smart card, then *U_a_* can guess the secret keywords and find the value of *α_i_.* *U_a_* finds *k_i_* = *M*_1_⨁*h*(*α_i_*||*MP_i_*). Let *U_a_* select random nonce *k_i_^#^* then modify the parameter *M*_1_ and *M*_2_ as *M*_1_***^#^*** = *k_i_^#^* ⨁ *h*(*α_i_*||*MP_i_*) and *M*_2_***^#^*** = *h*(*α_i_*||*MP_i_*||*k*_i_^#^||*T*_1_**^#^**). After that, *U_a_* sends the modified message {*M*_1_***^#^***, *M*_2_***^#^*,** *ID_i_*, *T*_1_***^#^***} to *GW*.
2. By gateway, after receiving the message {*M*_1_***^#^***, *M*_2_***^#^***, *ID_i_*, *T*_1_***^#^***}, the gateway examines the legality of the time stamp by figuring out|*T*_1_***^#^*** *− T_c_*| < ∆*T.* If the legality stays, then there are further attempts to figure out the subsequent steps; if not, a rejection message drops to the user *U_i_*.
3. The gateway computes k_i_^#*^ = *M*_1_**^#^** ⨁ *h*(*α_i_*||*h*(*PW_i_*)) and then computes *M*_2_^*^ = *h* (*α_i_*||*h*(*PW_i_*)||k_i_^#*^||*T*_1_**^#^**) and checks whether *M*_2_^*^ = ? *M*_2_***^#^***. If it holds, then the gateway authenticates the user *U_i;_* if not, it sends a rejection message to the user.
4. *GW* computes *ϒ_ij_* = *h*(*α_i_*||*β_j_*||*ID_i_*||*ID_sj_*), *M*_3_ = *α_i_* ⨁ *ϒ_ij_*, and *M*_4_ = *h*(*ϒ_ij_*||*M*_3_||*ID_i_*||*T*_2_) and sends {*M*_3_, *M*_4_, *ID_i_*,*T*_2_} to the user.
5. Adversary *U_a_* intercepts the message {*M*_3_, *M*_4_, *ID_i_*, *T*_2_**}** and computes the value *ϒ_ij_* = *M*_3_ ⨁ *α_i_* and changes the gateway’s time stamp and parameter *M*_4_ as *M*_4_**^#^.**

Now *U_a_* delivers {*M*_3_, *M*_4_^#^, *ID_i_*, *T*_2_^#^**}** to the user *U_i._* After receiving {*M*_3_, *M*_4_^#^, *ID_i_*, *T*_2_^#^**},** the user checks whether |*T*_2_***^#^*** − *T_c_*| < ∆*T* and then computes *ϒ_ij_* = *α_i_* ⨁ *M*_3_ and *M*_4_^*^ = *h*(*ϒ_ij_*||*M*_3_||*ID_i_*||*T*_2_*^#^*) and checks whether *M*_4_^*^ =? *M*_4_^#^. If it holds, then *GW* verification by the user holds; otherwise, abort the process.

6. When a message is sent at time *T*_2_ to the user *U_i_*, *GW* immediately computes *M*_5_ = *k_i_^#^* ⨁ *h*(*β_j_*||*ID_sj_*), *M*_6_ = *β_j_* ⨁ *ϒ_ij_*, and *M*_7_ = *h*(*ϒ_ij_*||*k_i_^#^*||*ID_sj_*||*T*_3_), then sends { *M*_5_, *M*_6_, *M*_7_, *ID_i_*, *ID_sj_*, *T*_3_} to *S_j_*.
7. The adversary *U_a_* intercepts the message {*M*_5_, *M*_6_, *M*_7_, *ID_i_*, *ID_sj_*,*T*_3_}. *U_a_* changes the time stamp and parameter as *M*_7_*^#^* = *h*(*ϒ_ij_*||*k_i_^#^*||*ID_sj_*||*T*_3_***^#^***). Now the adversary *U_a_* sends the message {*M*_5_, *M*_6_, *M*_7_*^#^*, *ID_i_*, *ID_sj_*, *T*_3_***^#^***} to *S_j_*.
8. When a message is received from the gateway, *S_j_* confirms whether |*T*_3_***^#^*** − *T_c_*| < ∆*T* and then computes *k_i_^#^* = *M*_5_ ⨁ *h*(*β_j_*||*ID_sj_*), *ϒ_ij_* = *β_j_* ⨁ *M*_6_, and *M*_7_^*^ = *h*(*ϒ_ij_*||*k_i_^#^*||*ID_Sj_*||*T*_3_**)** and checks whether *M*_7_^*^ =? *M*_7_^#^. If it holds, then the gateway is certified through the sensor node; if not, the sensor node sends a failure text to the gateway.
9. Once the gateway verification is completed, *S_j_* sensor node picks a random number *k_j_* and calculates the session key as *SK* = *h*(*k_i_^#^* ⨁ *k_j_*).
10. *S_j_* computes *M*_8_ = *k_j_* ⨁ *ϒ_ij_* and *M*_9_ = *h*(*k_j_*||*ID_Sj_*||*T*_4_) then transmits {*M*_8_, *M*_9_, *ID_i_*, *ID_sj_*, *T*_4_} to the user *U_i_*.
11. The adversary intercepts the message {*M*_8_, *M*_9_, *ID_i_*, *ID_sj_*, *T*_4_}. *U_a_* computes *k_j_* = *M*_8_ ⨁ *ϒ_ij_*, *M*_9_^*^ = *h*(*k_j_*||*ID_Sj_*||*T*_4_) and checks whether *M*_9_ = ? *M*_9_^*^. The adversary *U_a_* computes the session key *SK* = *h*(*k_i_^#^* ⨁ *k_j_*). Now U_a_ chooses random number *k_j_^#^* and computes *M*_8_*^#^* = *k_j_^#^* ⨁ *ϒ_ij_* and *M*_9_*^#^* = *h*(*k_j_^#^*||*ID_Sj_*||*T*_4_*^#^*). *U_a_* transmits the message {*M*_8_*^#^*, *M*_9_***^#^***, *ID_i_*,*ID_sj_*,*T*_4_***^#^***} to the user *U_i_*.
12. Once the message is received from sensor node *S_j_*, the user confirms the legality of the stamp |*T*_4_***^#^*** *− T_c_|* < ∆*T*. The user examines the effectiveness of the sensor node by figuring out its own version of k_j_^#*^ = *M*_8_*^#^* ⨁*ϒ_ij_* and *M*_9_^#*^ = *h*(k_j_^#*^||*ID_Sj_*||*T*_4_***^#^***) and confirms whether *M*_9_*^#^* = ? *M*_9_^#*^. If it holds, then it calculates the session key as *SK* = *h*(*k_i_* ⨁ *k_j_^#^*).

Two session keys are established here: one is between the user and adversary *SK* = *h*(*k_i_* ⨁ *k_j_^#^*). The second is *SK* = *h*(*k_i_^#^* ⨁ *k_j_*), which is between the sensor node and the adversary. The adversary makes a fool of both the user and the sensor node by behaving like a middleman.

## 5. Proposed Scheme

**Figure 5:**
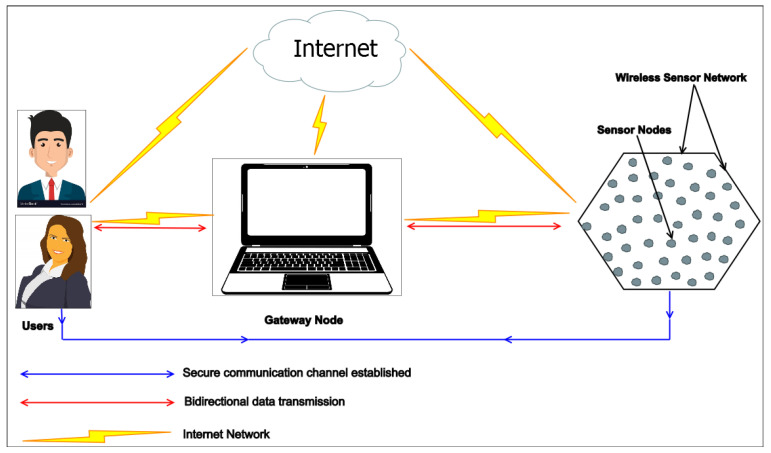
Sensors Enabled IoT Network.

Here, we propose an enhanced user authentication and key agreement scheme for WSNs tailored for IoT. This protocol is divided into four phases: registration, login, authentication and key agreement, and password change. Our scheme sorts out all the identified failures of Singh et al.’s scheme. The architecture of the sensors-enabled IoT network is shown in Figure 5. It depicts that the gateway node facilitates the establishment of a secure communication channel between the user and the sensor node. 

### 5.1. Registration

Here we split the phase into two sub-phases.

#### 5.1.1. Sensor Registration

Each sensor node *S_j_* has its identity *ID_sj_*. This section is performed by the *GW* offline before the use of sensor nodes in the target area. It contains the following steps:

- For each sensor node *S_j_*, the *GW* chooses an uncommon identity *ID_sj_*;
- The gateway node computes a common secret key between *GW* and *S_j_*

*K_GW-Sj_* = *h*(*ID_sj_*||*K_GW_*)

**Figure 6:**
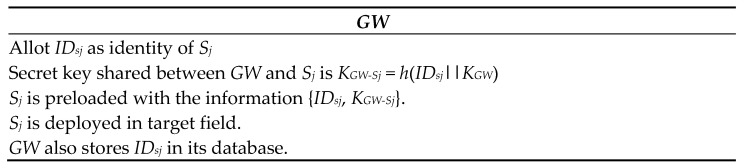
Sensor node pre-deployment phase.

Ultimately, every sensor node *S_j_* which is used in the target area is preloaded with the information {*ID_sj_*, *K_GW-Sj_*}, and *GW* also stores *ID_sj_* in its database. This phase is shown in Figure 6.

#### 5.1.2. User Registration

**Figure 7:**
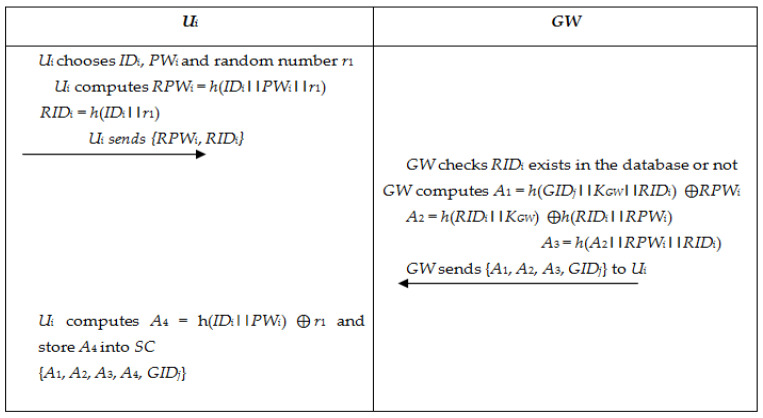
Registration phase between *U_i_* and *GW*.

In this section, a lawful user *U_i_* wishes to register with the *GW*. As a way to register to the *GW*, the user *U_i_* wishes to execute the steps which are given below and shown in Figure 7.

Step-1: User *U_i_* selects *ID_i_*, *PW_i_*, and random number *r*_1_. *U_i_* calculates

*RPW_i_* = *h*(*ID_i_||PW_i_||r*_1_)

*RID_i_* = *h*(*ID_i_||r*_1_)

Now *U_i_* forwards the registration request message {*RPW_i_*, *RID_i_*} to *GW* via a safe channel.

Step-2: *GW* investigates whether *RID_i_* exists in the database. If it exists, then *GW* forwards a rejection notification to *U_i_*. If not, *GW* saves *RID_i_* in the database and computes.

*A*_1_*= h*(*GID_j_||K_GW_||RID_i_*) ⨁ *RPW_i_*

*A*_2_*= h*(*RID_i_||K_GW_*) ⨁ *h*(*RID_i_||RPW_i_*)

*A*_3_*= h*(*A*_2_||*RPW_i_||RID_i_*)

*GW* stores {*A*_1_, *A*_2_, *A*_3_, *GID_j_*} into *SC* and sends *SC* to *U_i_* by a private channel.

Step-3: *U_i_* computes *A*_4_ = *h*(*ID_i_*||*PW_i_*) ⨁ *r*_1_ and stores *A*_4_ into *SC* {*A*_1_, *A*_2_, *A*_3_, *A*_4_, GID_j_}.

### 5.2. Login Phase

Subsequent to the completion of the registration phase, the user can contact a sensor node by the *GW*. Comprehensive steps are given underneath.

Step-1: User *U_i_* enters its smart card into the terminal and loads *ID_i_* and *PW_i_*.

Step-2: *SC* computes *r*_1_ = *A*_4_ ⨁ *h*(*ID_i_*||*PW_i_*);

And RPW_i_ = *h*(ID_i_||PW_i_||r_1_), RID_i_ = *h*(ID_i_||r_1_);

And checks *A*_3_ = ? *h*(*A*_2_||*RPW_i_||RID_i_*);

Step-3: If they do not match, then the login process will be canceled.

Step-4: If the password entered by the user was correct, then it selects the random number *r_u_* and required sensor *ID_sj_* and computes

*B*_1_ = *A*_1_ ⨁ *RPW_i_* = *h*(*GID_j_||K_GW_||RID_i_*)

*B*_2_ = *B*_1_ ⨁ *r_u_*

*B*_3_ = *h*(*GID_j_||ID_sj_||B*_1_||*RID_i_||r_u_||T*_1_)

Finally, the message *M*_1_ = {*B*_2_, *B*_3_, *GID_j_*, *RID_i_*, *ID_sj_*, *T*_1_} is sent to *GW*, where *T*_1_ is an ongoing time stamp.

### 5.3. Authentication and Key Agreement Phase

**Figure 8:**
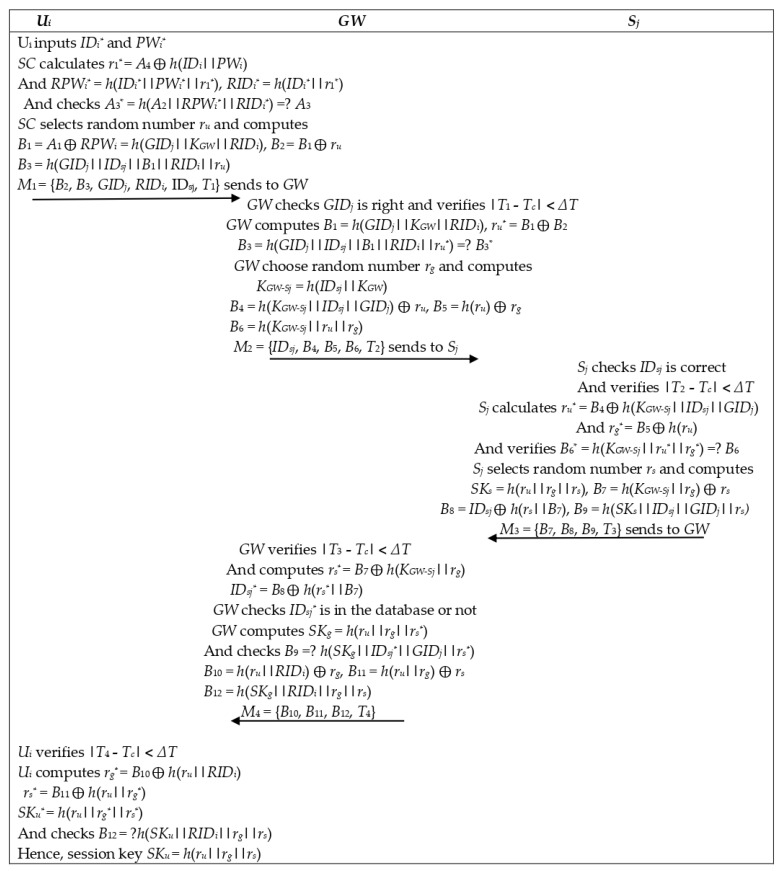
Authentication and key agreement phase.

Subsequent to accepting the login request message by the *GW* from *U_i_*, subsequent steps are accomplished for mutual authentication and key establishment. The login and authentication phases are shown in Figure 8.

Step-1: Firstly, *GW* checks if *GID_j_* is right. After that, *GW* verifies the validity of the timestamp. If |*T*_1_ − *T_c_|* < ∆*T* holds, then *GW* proceeds to further steps; otherwise, abort the process. *GW* computes

*B*_1_ = *h*(*GID_j_||K_GW_||RID_i_*)

r_u_^*^ = *B*_1_ ⨁ *B*_2_

Then checks *B*_3_^*^ = h(*GID_j_||ID_sj_||B*_1_||RID_i_||r_u_^*^||T_1_) = ? *B*_3_

If this does not hold, the user account *RID_i_* will be locked. Otherwise, *GW* searches for *ID_sj_* from the database, chooses a random number *r_g_*, and calculates

*K_GW-Sj_* = *h*(*ID_sj_||K_GW_*)

*B*_4_ = *h*(*K_GW-Sj_||ID_sj_||GID_j_*) ⨁ *r_u_*

*B*_5_*= h*(*r_u_*) ⨁ *r_g_*

*B*_6_ = *h*(*K_GW-Sj_||r_u_||r_g_||T*_2_)

*GW* sends the message *M*_2_ = {*ID_sj_*, *B*_4_, *B*_5_, *B*_6_, *T*_2_} to *S_j_*, where *T*_2_ is *GW* ongoing time stamp.

Step-2: Subsequent to accepting the message, *S_j_* first checks if *ID_sj_* is correct; after that, *S_j_* verifies the legality of the time stamp. If |*T*_2_ − *T_c_|* <∆*T* holds, then *S_j_* proceeds to further steps; otherwise, it sends a rejection message to *GW*.

*S_j_* calculates r_u_^*^ = *B*_4_ ⨁ *h*(*K_GW-Sj_*||*ID_sj_*||*GID_j_*)

and r_g_^*^ = *B*_5_ ⨁ *h*(*r_u_*)

and verifies *B*_6_^*^ = *h*(*K_GW-Sj_*||r_u_^*^||r_g_^*^||T_2_) = ? *B*_6_

If the equation is right, *S_j_* selects *r_s_* and computes

*SK_s_* = *h*(*r_u_||r_g_||r_s_*)

*B*_7_ = *h*(*K_GW-Sj_||r_g_*) ⨁ *r_s_*

*B*_8_ = *ID_sj_* ⨁ *h*(*r_s_||B*_7_)

*B*_9_ = *h*(*SKs||ID_sj_||GID_j_||r_s_||T*_3_)

Now *S_j_* sends *M*_3_ = {*B*_7_, *B*_8_, *B*_9_, *T*_3_} to *GW*

Step-3: Subsequent to accepting the message, *GW* verifies the legality of the time stamp. If |*T*_3_ − *T_c_|* < ∆*T* holds, then *GW* goes ahead to further steps; if not, abort the process. 

*GW* computes r_s_^*^ = *B*_7_ ⨁ *h*(*K_GW-Sj_||r_g_*)

ID_sj_^*^ = *B*_8_ ⨁ *h*(r_s_^*^||B_7_)

Then investigates ID_sj_^*^ in the database. If it does not occur, then *GW* stops the process; otherwise, *GW* checks *B*_9_^*^ = *h*(SK_g_||ID_sj_^*^||GID_j_||r_s_^*^||T_3_) =? *B*_9_

*GW* computes *SK_g_* = *h*(*r_u_||r_g_||r_s_*)

*B*_10_ = *h*(*r_u_||RID_i_*) ⨁ *r_g_*

*B*_11_ = *h*(*r_u_||r_g_*) ⨁ *r_s_*

*B*_12_ = *h*(*SK_g_||RID_i_||r_g_||r_s_||T*_4_)

*GW* sends the message *M*_4_ = {*B*_10_, *B*_11_, *B*_12_, *T*_4_} to *U_i_*.

Step-4: Subsequent to accepting the message, *U_i_* investigates the legality of the time stamp. If |*T*_4_ − *T_c_|* < ∆*T* holds, then *U_i_* proceeds to further steps; otherwise, it stops the process.

*U_i_* computes r_g_^*^ = *B*_10_ ⨁ *h*(*r_u_||RID_i_*)

r_s_^*^ = *B*_11_ ⨁ *h*(*r_u_*||*r_g_*)

SK_u_^*^ = *h*(*r_u_*||r_g_^*^||r_s_^*^)

Then checks *B*_12_^*^ = *h*(SK_u_^*^||RID_i_||r_g_^*^||r_s_^*^||T_4_) = ? *B*_12_

Hence, Session key *SK_u_* = *h*(*r_u_||r_g_||r_s_*)

### 5.4. Password Change Phase

Step-1: User *U_i_* inserts its *SC* into the terminal and inputs his/her *ID_i_* and *PW_i_*.

*SC* computes *r*_1_ = *A*_4_ ⨁ *h*(*ID_i_||PW_i_*)

*RPW_i_* = *h*(*ID_i_||PW_i_||r*_1_) and *RID_i_* = *h*(*ID_i_||r*_1_)

Chooses a random number *r_u_* and calculates *B*_1_, *B*_2_, and *B*_13_ = *h*(*GID_j_*||*B*_1_||*RID_i_||r_u_||T*_5_). Finally, it sends *M*_5_ = {*GID_j_*, *RID_i_*, *B*_2_, *B*_13_, *T*_5_} with to *GW*.

Step-2: *GW* investigates legality of time stamp *T*_5_ after that calculates *B*_1_, *r_u_*, and checks *B*_13_ =? *h*(*GID_j_*||*B*_1_||*RID_i_||r_u_||T*_5_)

*GW* computes *B*_14_ = *h*(*GID_j_*||*K_GW_||RID_i_*) ⨁ *h*(*r_u_*||*RID_i_*)

*B*_15_ = *h*(*RID_i_||GID_j_||B*_14_||*T*_6_)

Finally, it sends *M*_6_ = {*B*_14_, *B*_15_} to *U_i_*.

Step-3: After receiving *M*_6_, *SC* checks the validity of time stamp and checks *B*_15_ = ? *h*(*RID_i_*||*GID_j_||B*_14_||*T*_6_). If so, *U_i_* inputs a new password *PW_i_^new^*, and the *SC* generates an arbitrary number *r*_1_*^new^*, and calculates

*RPW_i_^new^* = *h*(*RID_i_||PW_i_^new^||r*_1_*^new^*)

*A*_1_*^new^* = *B*_14_ ⨁ *h*(*r_u_||RID_i_*) ⨁ *h*(*ID_i_||PW_i_^new^||r*_1_)

*A*_2_*^new^*= *A*_2_ ⨁ *h*(*RID_i_||RPW_i_*) ⨁ *h*(*RID_i_||RPW_i_^new^*)

*A*_3_*^new^ = h*(*A*_2_*^new^||RPW_i_^new^||RID_i_*)

Finally, the *SC* replaces {*A*_1_, *A*_2_, *A*_3_} with {*A*_1_*^new^*, *A*_2_*^new^*, *A*_3_*^new^*}.

## 6. Security Analysis

Here, we discuss the security of our scheme formally as well as informally.

### 6.1. Informal Security Analysis

#### 6.1.1. Insider Attack Resistance

When a user sends a registration request message {*RPW_i_*, *RID_i_*} to *GW*, an insider of *GW* obtains these secret values. Moreover, the insider obtains the parameters stored in *SC* {*A*_1_, *A*_2_, *A*_3_, *A*_4_, *GID_j_*}. To find the random number *r*_1_, the adversary needs to guess ID_i_^*^ and PW_i_^*^ simultaneously because *A*_4_ = *h*(*ID_i_*||*PW_i_*) ⨁ *r*_1_. However, the probability of guessing ID_i_^*^ and PW_i_^*^ simultaneously is negligible. The adversary cannot find the random number *r*_1_ and cannot guess ID_i_^*^ and PW_i_^*^. The adversary cannot verify whether *RPW_i_* =? RPW_i_^*^ where *RPW_i_* = *h*(*ID_i_*||*PW_i_||r*_1_). Hence, an insider cannot guess the user’s password.

#### 6.1.2. Offline Password Guessing Resistance

The adversary obtains the *SC* {*A*_1_, *A*_2_, *A*_3_, *A*_4_, *GID_j_*} and obtains the parameter stored in it. From *A*_4_ = *h*(*ID_i_*||*PW_i_*) ⨁ *r*_1_, the adversary knows only *A*_4_. To find the random number *r*_1_, the adversary needs to guess ID_i_^*^ and PW_i_^*^ simultaneously. However, the probability of guessing ID_i_^*^ and PW_i_^*^ simultaneously is negligible. In other equations, *A*_1_ = *h*(*GID_j_*||*K_GW_||RID_i_*) ⨁ *RPW_i_*, *A*_2_ = *h*(*RID_i_*||*K_GW_*) ⨁ *h*(*RID_i_*||*RPW_i_*), and *A*_3_ = *h*(*A*_2_||*RPW_i_||RID_i_*) the password is used implicitly. From these equations, if the adversary wants to guess the password *PW_i_* then he/she needs to know *ID_i_* and the random number *r*_1_. In these equations, the random number is not used in any of the equations, and *ID_i_* is used implicitly. The adversary cannot guess the password from these equations. Thus the proposed scheme is safe against offline password-guessing attacks.

#### 6.1.3. Identity Guessing Resistance

The correct value of the user identity (*ID_i_*) is only known to *U_i_*, and the gateway node saves *RID_i_* = *h*(*ID_i_||r*_1_), in which *ID_i_* concatenates with the random number *r*_1_. The user does not use his/her identity for login or for authentication. In the whole scheme, the user identity is used only inside *A*_4_ = *h*(*ID_i_*||*PW_i_*) ⨁ *r*_1_. It is not possible for the adversary to find the random number *r*_1_, and it can be easily seen that the adversary needs to accurately guess the *PW_i_* and *ID_i_* simultaneously, but at the same time, it is not possible. Hence, our scheme does not suffer from identity-guessing attacks.

#### 6.1.4. User Forgery Resistance

If the adversary wants to forge the user, then the adversary needs to forge *M*_1_ = {*B*_2_, *B*_3_, *GID_j_*, *RID_i_*, *ID_sj_*, *T*_1_}; the adversary must calculate *B*_2_, *B*_3_. However, in the calculation of *B*_2_ = *B*_1_ ⨁ *r_u_* and *B*_3_ = *h*(*GID_j_*||*ID_sj_||B*_1_||*RID_i_||r_u_||T*_1_), *B*_1_= *h*(*GID_j_*||*K_GW_*||*RID_i_*) is required. In the calculation of *B*_1_, gateway node secret key *K_GW_* is required. Thus it is not desirable for an adversary to forge a user. The user *U_i_* and our scheme are secure against user forgery attacks.

#### 6.1.5. Sensor Capture Resistance

If the adversary captured some sensors, other than *S_j_*, which communicate with *U_i_*, the adversary could not forge *M*_3_ = {*B*_7_, *B*_8_, *B*_9_, *T*_3_} since *K_GW-Sj_* is used to construct *B*_7_ = *h*(*K_GW-Sj_*||*r_g_*) ⨁ *r_s_*. The sensors are captured by the adversary and have no association with *K_GW-Sj_*. So, even though other sensors are seized, *U_a_* cannot execute this attack successfully.

#### 6.1.6. Gateway Forgery Attack

To apply this attack, the adversary wants to forge *M*_2_ or *M*_4_, where *M*_2_ = {*ID_sj_*, *B*_4_, *B*_5_, *B*_6_, *T*_2_} and *M*_4_ = {*B*_10_, *B*_11_, *B*_12_, *T*_4_}. To forge the message *M*_2_, adversary must calculate *B*_4_, *B*_5_, and *B*_6_ where *B*_4_ = *h*(*K_GW-Sj_*||*ID_sj_*||*GID_j_*) ⨁ *r_u_*, *B*_5_ = *h*(*r_u_*) ⨁ *r*_g_, and *B*_6_ = *h*(*K_GW-Sj_*||*r_u_*||*r_g_*||*T*_2_). However, in the calculation of *B*_4_ and *B*_6_, *K_GW-Sj_* shared secret key between *GW* and sensor node is required. To forge the message *M*_4_, he/she must calculate *B*_10_, *B*_11_, and *B*_12_ where *B*_10_ = *h*(*r_u_*||*RID_i_*) ⨁ *r*_g_, *B*_11_ = *h*(*r_u_*||*r_g_*) ⨁ *r_s_*, and *B*_12_ = *h*(*SK_g_*||*RID_i_*||*r_g_*||*r_s_*||*T*_4_). However, it is not possible to calculate *B*_10_, *B*_11_, and *B*_12_ because random numbers and session keys are required. It is not possible to forge a gateway node. Hence, the proposed scheme is safe against gateway forgery attacks.

#### 6.1.7. De-synchronization Resistance

De-synchronization is a very big security issue in WSNs. Our scheme includes a random number mechanism to assure the originality of interchanged messages and also uses a timestamp mechanism. In each session of our scheme, random numbers *r_u_, *r*_g_*, and *r_s_* are generated by *U_i_*, *GW*, and *S_j,_* respectively. Hence, our scheme is free from de-synchronization problems. 

#### 6.1.8. No Adversarial Session Key Agreement

To change the session key, the adversary needs to change any of the random numbers *r_u_*, *r_g_*, and *r_s_*.

When the message *M*_1_ = {*B*_2_, *B*_3_, *GID_j_*, *RID_i_*, *ID_sj_*, *T*_1_} is sent to *GW*, if the adversary wants to agree on the session key with *GW* and *S_j_*, then *U_a_* selects a random number *r_u_*. Now, the adversary needs to calculate *B*_2_ and *B*_3_. *B*_2_ = *B*_1_ ⨁ *r_u_* and *B*_1_ = *A*_1_ ⨁ *RPW_i_* = *h*(*GID_j_*||*K_GW_||RID_i_*). In *B*_1_ = *A*_1_ ⨁ *RPW_i_*, *U_a_* cannot calculate *B*_1_ from this because, in Section 5.2, the adversary cannot guess the user’s password. In *B*_1_ = *h*(*GID_j_*||*K_GW_||RID_i_*), *K_GW_* is *GW’s* secret key which is only known by *GW*. The adversary cannot calculate *B*_1_ with this. In the calculation of *B*_3_ = *h*(*GID_j_*||*ID_sj_||B*_1_||*RID_i_||r_u_||T*_1_), he/she must know *B*_1_ and random number *r_u_*. As discussed above, we conclude that *U_a_* cannot calculate *B*_1_ and *U_a_* also cannot calculate *B*_3_. In message *M*_1_, the adversary cannot make any type of changes.

When message *M*_2_ = {*ID_sj_*, *B*_4_, *B*_5_, *B*_6_, *T*_2_} is sent to *S_j_*, If the adversary wants to change the session key, then *U_a_* selects a random number r_g_^*^. Now, the adversary needs to calculate *B*_5_ and *B*_6_ where *B*_5_ = *h*(*r_u_*) ⨁ *r_g_*. *U_a_* does not know the random number *r_u_* selected by *U_i_*, then he/she cannot calculate *B*_5_. In *B*_6_ = *h*(*K_GW-Sj_*||*r_u_*||*r_g_*||*T*_2_), *K_GW-Sj_* is a shared secret key between *GW* and *S_j_*. The adversary cannot calculate *B*_6_. In message *M*_2_, the adversary cannot make any type of change.

When message *M*_3_ = {*B*_7_, *B*_8_, *B*_9_, *T*_3_} is sent to *GW*, if *U_a_* wants to change the session key, then *U_a_* selects a random number r_s_^*^. Now, the adversary needs to calculate *B*_7_, *B*_8_, and *B*_9_. The adversary needs to know *K_GW-Sj_* and *r_g_* to calculate *B*_7_ = *h*(*K_GW-Sj_*||*r_g_*) ⨁ *r_s_*, but *K_GW-Sj_* shares the secret key only between *GW* and *S_j_*, so the adversary cannot calculate *B*_7_. To calculate *B*_8_ = *ID_sj_* ⨁ *h*(*r_s_*||*B*_7_), *U_a_* needs to know *B*_7_. Above, we conclude that *U_a_* cannot calculate *B*_7_ and the adversary cannot calculate *B*_8_. *U_a_* needs to know *SKs* in order to calculate *B*_9_ = *h*(*SKs*||*ID_sj_||GID_j_||r_s_||T*_3_) where *SKs* = *h*(*r_u_*||*r_g_||r_s_*). *U_a_* does not know the random numbers *r_u_* and *r_g_*, and the adversary cannot calculate *B*_9_.

Hence, our proposed scheme is safe from adversarial session key agreement.

#### 6.1.9. Man-In-The-Middle Attack

To apply a man-in-the-middle attack, the adversary works as a middleman between the user and the sensor node. In this attack, one session key is conducted between the user and adversary, and another session key is established between the adversary and sensor node. Both the user and the sensor node believe they are communicating with each other, but in this attack, both are communicating with the adversary. 

When message *M*_1_ = {*B*_2_, *B*_3_, *GID_j_*, *RID_i_*, *ID_sj_*, *T*_1_} is sent to *GW,* then the adversary intercepts it and tries to find random number *r_u_* where *r_u_* = *B*_2_ ⨁ *B*_1_ and *B*_1_ = *A*_1_ ⨁ *RPW_i_* = *h*(*GID_j_*||*K_GW_||RID_i_*). The adversary does not know *RPW_i_* and *K_GW_*. As a result, he/she cannot find *r_u_* and cannot able to apply this attack at this end.

Similarly, when the sensor node sends message *M*_3_ = {*B*_7_, *B*_8_, *B*_9_, *T*_3_} to *GW,* then the adversary needs to know the random number *r_s_* = *B*_7_ ⨁ *h*(*K_GW-Sj_*||*r_g_*). However, the adversary does not know *K_GW-Sj_* and *r_g_*. So he/she cannot be able to find the random number *r_s_*.

Hence, our proposed scheme is safe from a man-in-the-middle attack. 

#### 6.1.10. Stolen Smart Card Resistance

Suppose *SC* of the user has been lost, then all the information stored in *SC* obtains by an adversary. In our proposed scheme *SC* has the parameters {*A*_1_, *A*_2_, *A*_3_, *A*_4_, *GID_j_*} where *A*_1_ = *h*(*GID_j_*||*K_GW_||RID_i_*) ⨁ *RPW_i_*, *A*_2_ = *h*(*RID_i_*||*K_GW_*) ⨁ *h*(*RID_i_*||*RPW_i_*), *A*_3_ = *h*(*A*_2_||*RPW_i_||RID_i_*), and *A*_4_ = *h*(*ID_i_*||*PW_i_*) ⨁ *r*_1_. However, without knowing (*ID_i_*, *r*_1_), *U_a_* cannot obtain the user’s password. An adversary cannot obtain any secret information from it. Hence our proposed protocol resists stolen smart card attacks.

#### 6.1.11. User Anonymity Provision

Our scheme protects *ID_i_* with *h*(*ID_i_*||*r*_1_). It also protects *PW_i_* with *h*(*ID_i_*||*PW_i_||r*_1_). Thus in order to obtain *ID_i_*, a random number *r*_1_ is needed, and to obtain *U_i_^’^s* password *U_i_^’^s* identity and random number *r*_1_ need to be known. Moreover, even if a stolen smart card is obtained by the adversary, *U_a_* cannot obtain *ID_i_* from *A*_4_ = *h*(*ID_i_*||*PW_i_*) ⨁ *r_1_* since *ID_i_* is protected by *h*(*ID_i_*||*PW_i_*) ⨁ *r*_1_. The adversary cannot find the identity and password of the user. This proves that our suggested protocol provides user anonymity.

#### 6.1.12. Mutual Authentication Provision

*GW* checks *B*_4_ = *h*(*K_GW-Sj_*||*ID_sj_*||*GID_j_*) ⨁ *r_u_* to verify *U_i_*, and *B*_9_ = *h*(*SK_g_*||*ID_sj_*||*GID_j_*||*r_s_*||*T*_3_) to verify *S_j_*, *S_j_* checks *ID_Sj_* and *B*_6_ = *h*(*K_GW-Sj_*||*r_u_*||*r_g_*||*T*_2_) to authenticate *GW* directly and *U_i_* indirectly. *U_i_* checks *B*_12_ = *h*(*SK_g_*||*RID_i_*||*r_g_*||*r_s_*||*T*_4_) to justify *GW* directly and *S_j_* indirectly. So, either pair of parties achieves mutual authentication.

#### 6.1.13. Password Updating/Changing Provision

Suppose a legitimate user has his/her smart card stolen. Suppose the information is acquired by the adversary who saves in *SC*. Suppose the adversary revealed the information which is saved in *SC*. To change the password, it is necessary for the adversary to know the existing password *PW_i_* verification. Moreover, it is not possible to find the old password because the password is protected with *RPW_i_* = *h*(*ID_i_*||*PW_i_||r*_1_). In this way, an adversary needs to reckon the existing password before updating another password.

### 6.2. Formal Security Analysis

Here, we do a formal security analysis of our scheme with the help of a random oracle model. In this section, we use the Real or Random (RoR) [35] model to prove that the proposed protocol is secure. In the RoR model, the attacker is given the right to query and uses the interactive question and answer with a random oracle to verify the security of the proposed scheme. There are two participants in the proposed protocol: $\Pi_{I}^{m}$ and $\Pi_{S}^{n}$ represent the m-thIoT device instance and the n-th trusted server instance respectively. In addition, for formal security analysis, we define the following query model for attacker A.

Execute(O): A by executing this query, he can intercept the messages transmitted by IoT devices and trusted server servers on the public channel, where $O=\left\{\Pi_{I}^{m},\Pi_{S}^{n}\right\}$.

Send(O, M):By executing the query, A can send message M to O and receive a response from O.

Hash(string):By executing the query, A can enter a string and return its hash value.

Test(O):A flips a coin c by executing this query. If c=1, A can obtain the correct session key. If c=0, A can obtain a random string with the same length as the session key.

**Theorem 1.**  *In the R.O.R model, suppose *A*can execute the queries of *Execute(O)*, *Send(O, M), Hash(string)*, and *Test(Z)*, the probability *P*of A breaking the protocol in polynomial time is: *$Adv_{A}^{P}\left(\varepsilon \right)\leq \frac{q_{h}^{2}}{\left|Hash\right|}+\frac{q_{P}^{2}}{\left|PUF\right|}+2Adv_{A}^{\Omega }\left(\varepsilon \right)$*. Here, *$q_{h}$*refers to the number of times the hash is executed, *$q_{p}$*refers to the number of times *PUF*is executed. *Hash *and *PUF*refer to scope space of hash function*H(·)*and *PUF*function *PUF(·). $Adv_{A}^{\Omega }\left(\varepsilon \right)$*represents the advantage of *A*cracking the symmetric cipher *Ω, *for a sufficiently small number *γ, *then *$Adv_{A}^{\Omega }\left(\varepsilon \right)<\gamma$.

**Proof.**  We defined five rounds of the game $GM_{0}-GM_{4}$ to simulate the attack process of A. In the process of proving, $Succ_{A}^{GM_{i}}\left(\varepsilon \right)$ represents the probability that A can win multiple rounds of the game, $Adv_{A}^{P}\left(\varepsilon \right)$ means that A can break the advantage of protocol. The proof steps are as follows:

$GM_{0}:\;$ In the ROR model, $GM_{0}$ game is a real attack on the authentication key exchange protocol proposed by A, and A flips the coin c at the beginning of the game. Therefore, we obtain the following results:

$$Adv_{A}^{P}\left(\varepsilon \right)=\left|2\mathrm{Pr}\left[Succ_{A}^{GM_{0}}\left(\varepsilon \right)\right]-1\right|$$

$GM_{1}$: With $GM_{0}$ being different from $GM_{1}$ by executing the Execute query, A can intercept the messages $\left\{h(ID_{A}),\;Auth_{req},\;TS_{1},\;h(ID_{A},TS_{1})\right\}$,$\left\{C_{A,i},\;P_{A,i},\;TS_{2},\;h(h(ID_{A}),C_{A,i},\;P_{A,i}),\;h(K_{A,i})\right\}$, and $\{P_{A,i},\;TS_{3},\;h(h(ID_{A}),TS_{3},K_{A,i},\;R_{A,i+1}),(R_{A,i+1}),\;{\left(R_{A,i+1}\right)}_{h(K_{A,i})}\}\;$transmitted on the public channel. Then, A will perform a Test query to calculate the session key $h(K_{A,i})$, but the message intercepted on the public channel cannot help A calculate SK. Therefore, the probability of A winning $GM_{1}$ by eavesdropping information will not increase. So we obtain:

$$\mathrm{Pr}\left[Succ_{A}^{GM_{1}}\left(\varepsilon \right)\right]=\mathrm{Pr}\left[Succ_{A}^{GM_{0}}\left(\varepsilon \right)\right]$$

$GM_{2}:$Different from $GM_{1}$, $GM_{2}$ adds Hash query and Send query. In the intercepted messages$\;\{C_{A,i},\;P_{A,i},\;TS_{2},\;h(h(ID_{A}),C_{A,i}),h(K_{A,i})\}$ and $\left\{P_{A,i},\;TS_{3},\;h(h(ID_{A}),TS_{3},\;R_{A,i+1}),(R_{A,i+1}),\;{\left(R_{A,i+1}\right)}_{h(K_{A,i})}\right\}$, the parameters $h(h(ID_{A}),C_{A,i}),h(K_{A,i})$,$h(K_{A,i})\;$and $\left\{h(h(ID_{A}),TS_{3},K_{A,i},\;R_{A,i+1}\right)$ are based on the one-way hash function. In addition, $h(K_{A,i})\;$is different in each communication; the hash function will not collide. Therefore, according to the birthday paradox [36], we can obtain

$$\left|\mathrm{Pr}\left[Succ_{A}^{GM_{2}}\left(\varepsilon \right)\right]-\mathrm{Pr}\left[Succ_{A}^{GM_{1}}\left(\varepsilon \right)\right]\right|\leq \frac{q_{h}^{2}}{2\left|Hash\right|}$$

$GM_{3}:$The difference between $GM_{3}$ and $GM_{2}$ is that $GM_{3}$ adds PUF query. A executes Send and PUF queries. Because the physical function PUF has security attributes. Therefore, we can obtain

$$|\mathrm{Pr}\left[Succ_{A}^{GM_{3}}\left(\varepsilon \right)\right]-\mathrm{Pr}\left[Succ_{A}^{GM_{2}}\left(\varepsilon \right)\right]|\leq \frac{q_{P}^{2}}{2\left|PUF\right|}$$

$GM_{4}:$In this game, A tries to crack the encrypted message ${\left(R_{A,i+1}\right)}_{h(K_{A,i})}$, In the security model in Section 3.2, it is defined that the attacker cannot crack the memory of the server, A cannot obtain $h(K_{A,i})$, so A cannot calculate $\left(R_{A,i+1}\right)$. According to the security of Ω symmetric encryption algorithm, we can obtain

$$|\mathrm{Pr}\left[Succ_{A}^{GM_{4}}\left(\varepsilon \right)\right]-\mathrm{Pr}\left[Succ_{A}^{GM_{3}}\left(\varepsilon \right)\right]|\leq Adv_{A}^{\Omega }\left(\varepsilon \right)$$

Because the probability of success and failure of A is equal, so the probability that A can guess the session key is

$$\mathrm{Pr}\left[Succ_{A}^{GM_{4}}\left(\varepsilon \right)\right]=1/2.$$

According to the above formula, we can obtain

$$\frac{1}{2}Adv_{A}^{P}\left(\varepsilon \right)=\left|\mathrm{Pr}\left[Succ_{A}^{GM_{0}}\left(\varepsilon \right)\right]-\frac{1}{2}\right|$$

$$=\left|\mathrm{Pr}\left[Succ_{A}^{GM_{0}}\left(\varepsilon \right)\right]-\mathrm{Pr}\left[Succ_{A}^{GM_{4}}\left(\varepsilon \right)\right]\right|$$

$$\leq \overset{3}{\underset{i=0}{\sum }}\left|\mathrm{Pr}\left[Succ_{A}^{GM_{i+1}}\left(\varepsilon \right)\right]-\mathrm{Pr}\left[Succ_{A}^{GM_{i}}\left(\varepsilon \right)\right]\right|$$

$$=\frac{q_{h}^{2}}{2\left|Hash\right|}+\frac{q_{P}^{2}}{2\left|PUF\right|}+Adv_{A}^{\Omega }\left(\varepsilon \right)$$

Therefore, the probability that A can crack the protocol is:

$$Adv_{A}^{P}\left(\varepsilon \right)\leq \frac{q_{h}^{2}}{\left|Hash\right|}+\frac{q_{P}^{2}}{\left|PUF\right|}+2Adv_{A}^{\Omega }\left(\varepsilon \right)$$

## 7. Comparisons with other Related Schemes

### 7.1. Comparison of Security and Functionality Features

**Figure 9:**
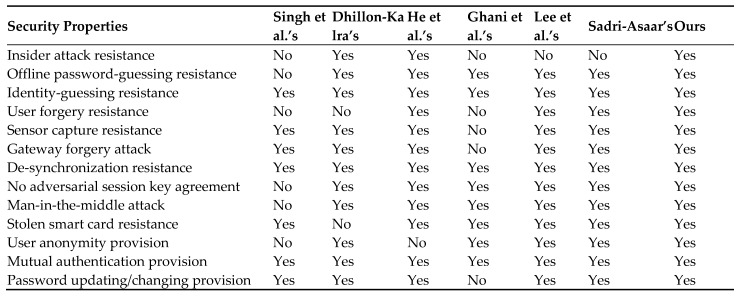
Comparison of security and functional features [15,19,21,22,27,34].

All the schemes [15,19,21,22,27,34] which are used in comparison suffer from security problems. The scheme in [34] suffers from insider attacks, offline password-guessing attacks, user forgery attacks, and session key disclosure attacks. This scheme does not provide user anonymity. The scheme in [15] suffers from user forgery attacks and stolen smart card attacks. The scheme in [19] does not provide user anonymity. The scheme in [21] suffers from insider attacks, user forgery attacks, sensor capture resistance, gateway forgery attacks, and password-changing provision. The scheme in [22,27] suffers from an insider attacks. Our proposed scheme resists all the security attacks which are mentioned in Figure 9. Our scheme provides functional features which cannot be seen in the related schemes [15,19,21,22,27,34].

### 7.2. Comparison of Computation Cost

**Figure 10:**
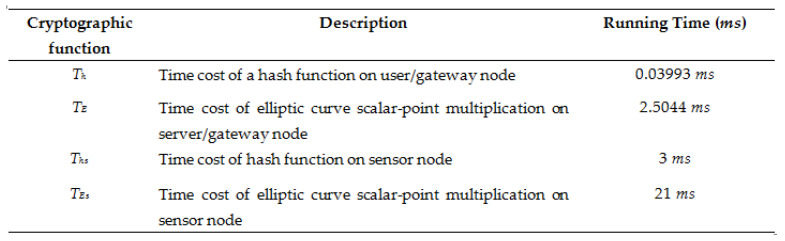
Cryptographic function and their description for computation cost.

**Figure 11:**
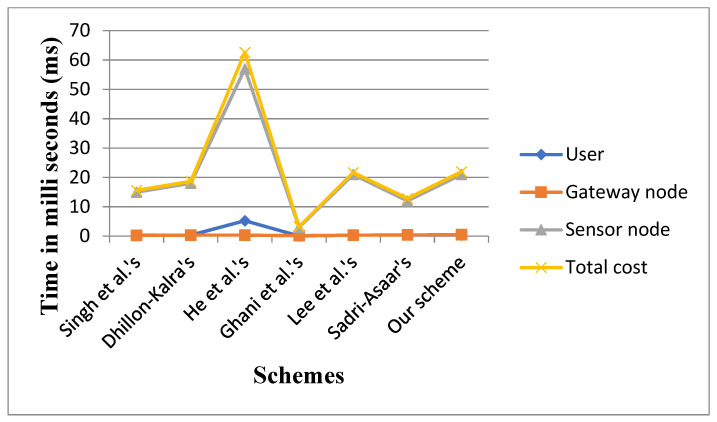
Comparison of computation cost.

Figure 10 defines cryptographic functions and their running time for comparison of computation cost. Figure 10 and Figure 11 together show the comparison of the computation cost of our scheme with schemes in [15,19,21,22,27,34].

**Figure 12:**
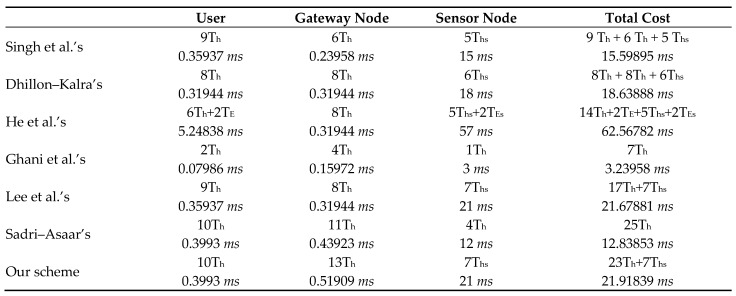
Comparison of computation cost [15,19,21,22,27].

On the user side, the scheme in [19] has the highest computation cost, while the scheme in [21] has the lowest computation cost. Protocol [22] and protocol [34] has equal computation cost. Our proposed scheme and the scheme in [27] have the second-highest computation cost. At the gateway node side, the scheme in [21] has the lowest computation cost, while the scheme in [15,19,22] has the same third-lowest computation cost. Our proposed scheme has the highest computation cost from the gateway node side. On the sensor node side, the scheme in [19] has the highest computation cost, while the scheme in [21] has the lowest computation cost. Our suggested scheme computation cost is slightly greater than the scheme in [22]. It is depicted in Figure 12 that the total computation cost of our scheme is slightly greater than the total computation cost of [22]. The scheme in [19] has the highest computation cost. The scheme in [21] has the lowest computation cost.

Our proposed scheme can be a little bit more costly than other related schemes, but our scheme has passed various hurdles in security checks which makes it user-friendly. Our scheme neither uses complex cryptographic operations nor does it add much computational load when compared to its counterparts. Moreover, the running time of an operation is directly proportional to the power consumption required to run that operation. Therefore, the proposed scheme is a power-efficient protocol.

### 7.3. Comparison of Communication Cost

**Figure 13:**
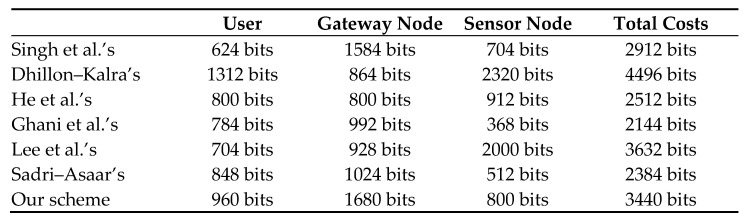
Comparison of communication cost [15,19,21,22,27,34].

**Figure 14:**
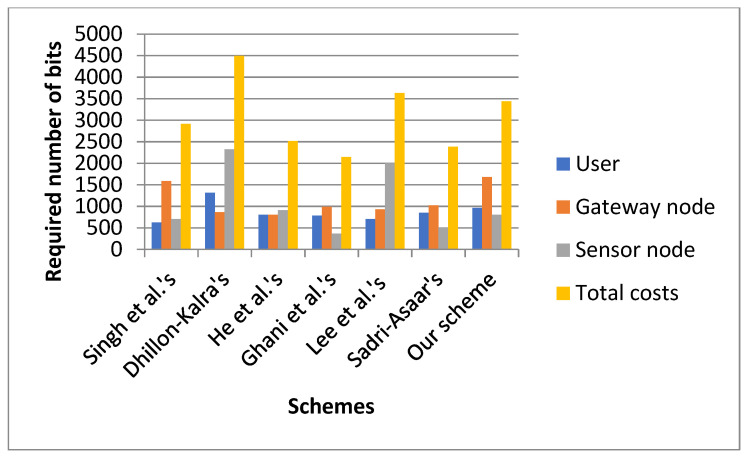
Comparison of communication cost.

In pursuance of comparing the communication cost of the suggested protocol with the relevant protocols, we consider the length of the elliptic curve scalar-point multiplication function, and the random number is 160 bits. We suppose the length of the identities, such as *ID_i_* and *ID_sj_*, and every coordinate point from the output of the elliptic curve scalar-point multiplication function is 80 bits. Let the output of the message authentication code be 160 bits. We suppose that each element is 160 bits in the elliptic curve group. Here, we have the hash (h(.)) function SHA2-256 with the output of length 256 bits. We consider the length of the timestamp as 32 bits. In Figure 13 and in Figure 14, we show the communication costs of the three entities in our proposed scheme and the related schemes [15,19,21,22,27,34].

From Figure 13 and Figure 14, we see that, on the user side, the communication cost of the protocol [34] is 624 bits which is the minimum, and Dhillon and Kalra’s scheme has the highest communication cost of 1312 bits. Our suggested scheme has the second-highest communication cost of 960 bits. At the gateway node side, our proposed scheme has the highest communication cost of 1680 bits, and the scheme in [19] has the lowest communication cost of 800 bits. At the sensor node aspect, our suggested scheme has a communication cost of 800 bits, while the protocol in [21] has the lowest communication cost of 368 bits. Dhillon and Kalra’s scheme has the highest communication cost of 2320 bits. The total communication cost of Dhillon and Kalra’s scheme is the highest, and in the proposed scheme, it is the third-highest. The scheme in [21] has the lowest communication cost.

## 8. Conclusions

We have analyzed Singh et al.’s authentication and key agreement scheme for WSNs and found some security pitfalls in it. Then we developed an improved authentication and key agreement scheme for WSNs tailored for IoT. The informal analysis of the proposed scheme indicates its resistance to various sorts of adversarial activities. The formal security of the proposed scheme with the RoR model further supports its security. In the end, we have compared the performance of our scheme with that of the related schemes. For the proposed scheme, we have tried to control the cost along with maintaining security.

## Data Availability

Not applicable.

## References

[1] Das M.L. (2009). Two-factor user authentication in wireless sensor networks. IEEE Trans. Wirel. Commun..

[2] Yeh H.-L., Chen T.-H., Liu P.-C., Kim T.-H., Wei H.-W. (2011). A Secured Authentication Protocol for Wireless Sensor Networks Using Elliptic Curves Cryptography. Sensors.

[3] Xue K., Ma C., Hong P., Ding R. (2013). A temporal-credential-based mutual authentication and key agreement scheme for wireless sensor networks. J. Netw. Comput. Appl..

[4] Turkanović M., Brumen B., Hölbl M. (2014). A novel user authentication and key agreement scheme for heterogeneous ad hoc wireless sensor networks, based on the Internet of Things notion. Ad Hoc Networks.

[5] Jiang Q., Ma J., Lu X., Tian Y. (2014). An efficient two-factor user authentication scheme with unlinkability for wireless sensor networks. Peer-to-Peer Netw. Appl..

[6] He D., Kumar N., Chilamkurti N. (2015). A secure temporal-credential-based mutual authentication and key agreement scheme with pseudo identity for wireless sensor networks. Inf. Sci..

[7] Kumari S., Li X., Wu F., Das A.K., Arshad H., Khan M.K. (2016). A user friendly mutual authentication and key agreement scheme for wireless sensor networks using chaotic maps. Futur. Gener. Comput. Syst..

[8] Jiang Q., Ma J., Wei F., Tian Y., Shen J., Yang Y. (2016). An untraceable temporal-credential-based two-factor authentication scheme using ECC for wireless sensor networks. J. Netw. Comput. Appl..

[9] Farash M.S., Turkanović M., Kumari S., Hölbl M. (2016). An efficient user authentication and key agreement scheme for heterogeneous wireless sensor network tailored for the Internet of Things environment. Ad Hoc Networks.

[10] Amin R., Biswas G. (2016). A secure light weight scheme for user authentication and key agreement in multi-gateway based wireless sensor networks. Ad Hoc Netw..

[11] Amin R., Islam S.H., Biswas G., Khan M.K., Leng L., Kumar N. (2016). Design of an anonymity-preserving three-factor authenticated key exchange protocol for wireless sensor networks. Comput. Netw..

[12] Chang C.-C., Hsueh W.-Y., Cheng T.-F. (2016). A Dynamic User Authentication and Key Agreement Scheme for Heterogeneous Wireless Sensor Networks. Wirel. Pers. Commun..

[13] Wu F., Xu L., Kumari S., Li X., Shen J., Choo K.-K.R., Wazid M., Das A.K. (2017). An efficient authentication and key agreement scheme for multi-gateway wireless sensor networks in IoT deployment. J. Netw. Comput. Appl..

[14] Wu F., Xu L., Kumari S., Li X. (2015). A new and secure authentication scheme for wireless sensor networks with formal proof. Peer-to-Peer Netw. Appl..

[15] Dhillon P.K., Kalra S. (2017). Secure multi-factor remote user authentication scheme for Internet of Things environments. Int. J. Commun. Syst..

[16] Amin R., Islam S.H., Biswas G., Khan M.K., Kumar N. (2018). A robust and anonymous patient monitoring system using wireless medical sensor networks. Futur. Gener. Comput. Syst..

[17] Srinivas J., Mishra D., Mukhopadhyay S. (2017). A Mutual Authentication Framework for Wireless Medical Sensor Networks. J. Med Syst..

[18] Li X., Niu J., Kumari S., Wu F., Sangaiah A.K., Choo K.-K.R. (2017). A three-factor anonymous authentication scheme for wireless sensor networks in internet of things environments. J. Netw. Comput. Appl..

[19] He J., Yang Z., Zhang J., Liu W., Liu C. (2018). On the security of a provably secure, efficient, and flexible authentication scheme for ad hoc wireless sensor networks. Int. J. Distrib. Sens. Netw..

[20] Gupta A., Tripathi M., Shaikh T.J., Sharma A. (2018). A lightweight anonymous user authentication and key establishment scheme for wearable devices. Comput. Networks.

[21] Ghani A., Mansoor K., Mehmood S., Chaudhry S.A., Rahman A.U., Saqib M.N. (2019). Security and key management in IoT-based wireless sensor networks: An authentication protocol using symmetric key. Int. J. Commun. Syst..

[22] Lee H., Kang D., Ryu J., Won D., Kim H., Lee Y. (2020). A three-factor anonymous user authentication scheme for Internet of Things environments. J. Inf. Secur. Appl..

[23] Mall P., Amin R., Obaidat M.S., Hsiao K.-F. (2021). CoMSeC++: PUF-based secured light-weight mutual authentication protocol for Drone-enabled WSN. Comput. Networks.

[24] Chen C.-M., Deng X., Gan W., Chen J., Islam S.K.H. (2021). A secure blockchain-based group key agreement protocol for IoT. J. Supercomput..

[25] Chen C.-M., Liu S. (2021). Improved Secure and Lightweight Authentication Scheme for Next-Generation IoT Infrastructure. Secur. Commun. Netw..

[26] Ali I., Chen Y., Ullah N., Kumar R., He W. (2021). An Efficient and Provably Secure ECC-Based Conditional Privacy-Preserving Authentication for Vehicle-to-Vehicle Communication in VANETs. IEEE Trans. Veh. Technol..

[27] Sadri M.J., Asaar M.R. (2021). An efficient hash-based authentication protocol for wireless sensor networks in Internet of Things applications with forward secrecy. Int. J. Commun. Syst..

[28] Rangwani D., Sadhukhan D., Ray S., Khan M.K., Dasgupta M. (2021). A robust provable-secure privacy-preserving authentication protocol for Industrial Internet of Things. Peer-to-Peer Netw. Appl..

[29] Nashwan S. (2021). An End-to-End Authentication Scheme for Healthcare IoT Systems Using WMSN. Comput. Mater. Contin..

[30] Tanveer M., Alkhayyat A., Khan A.U., Kumar N., Alharbi A.G. (2022). REAP-IIoT: Resource-Efficient Authentication Protocol for the Industrial Internet of Things. IEEE Internet Things J..

[31] Kumar V., Kumar R., Jangirala S., Kumari S., Kumar S., Chen C.-M. (2022). An Enhanced RFID-Based Authentication Protocol using PUF for Vehicular Cloud Computing. Secur. Commun. Networks.

[32] Wu T.-Y., Guo X., Chen Y.-C., Kumari S., Chen C.-M. (2022). SGXAP: SGX-Based Authentication Protocol in IoV-Enabled Fog Computing. Symmetry.

[33] Li Z., Miao Q., Chaudhry S.A., Chen C.-M. (2022). A provably secure and lightweight mutual authentication protocol in fog-enabled social Internet of vehicles. Int. J. Distrib. Sens. Netw..

[34] Singh A., Awasthi A.K., Singh K. (2016). Cryptanalysis and Improvement in User Authentication and Key Agreement Scheme for Wireless Sensor Network. Wirel. Pers. Commun..

[35] Canetti R., Goldreich O., Halevi S. The random oracle methodology, revisited (preliminary version). Proceedings of the Thirtieth Annual ACM Symposium on Theory of Computing.

[36] Boyko V., MacKenzie P., Patel S. (2000). Provably secure password-authenticated key exchange using Diffie-Hellman. Advances in Cryptology—EUROCRYPT 2000.

